# Efficacy and safety of the S1PR modulator etrasimod in the treatment of moderately to severely active ulcerative colitis during the induction phase: a systematic review and meta-analysis of randomized controlled trials

**DOI:** 10.3389/fphar.2024.1420455

**Published:** 2024-09-09

**Authors:** Jingyue Qiu, Jiakuo Liu, Kexin Cai, Ting Xu, Wenwen Liu, Fei Lin, Ning Shi

**Affiliations:** ^1^ Pharmaceutical Department, PLA Strategic Support Force Medical Center, Beijing, China; ^2^ Pharmaceutical Department, PLA Rocket Force Medical Center, Beijing, China; ^3^ Shandong Provincial Center for ADR Monitoring, Jinan, Shandong, China; ^4^ Department of Pharmacy, The First Affiliated Hospital of Chengdu Medical College, Chengdu, China; ^5^ Clinical Medical College, Chengdu Medical College, Chengdu, China

**Keywords:** S1PR modulator, etrasimod, ulcerative colitis, auto-immune disease, meta-analysis

## Abstract

**Background:**

The study aims to assess the efficacy and safety of the recently approved S1PR modulator etrasimod in adults with ulcerative colitis during the induction phase through meta-analysis.

**Methods:**

A systemic search was performed for randomized controlled trials evaluating the efficacy and safety of the S1PR modulator etrasimod using electronic databases PubMed, Embase, the Cochrane Library, Clinical Trials, and the International Clinical Trials Registry Platform. Three studies with 943 patients met the inclusion criteria and were included in this analysis. The study’s primary endpoint was the proportion of patients who achieved clinical remission at week 12. Key secondary endpoints included the proportion of patients with clinical response, endoscopic improvement, and histologic remission. The incidence of adverse effects (AEs), serious AEs (SAEs), and AE-related treatment discontinuation were statistically analyzed to determine the safety of etrasimod.

**Results:**

This study revealed that etrasimod is superior to placebo at the primary endpoint clinical remission (OR = 3.09, 95% CI: 2.04–4.69), as well as at the secondary endpoints clinical response (OR = 2.56, 95% CI: 1.91–3.43), endoscopic improvement (OR = 2.15, 95% CI: 1.51–3.05), and histologic remission (OR = 3.39, 95% CI: 2.03–5.68). The proportion of patients with TEAE (OR = 1.34, 95% CI: 1.01–1.78) and SAE (OR = 0.77, 95% CI: 0.41–1.43) was similar between the etrasimod and placebo groups. Patients receiving etrasimod had slightly higher odds of experiencing headache (OR = 2.07, 95% CI: 1.01–4.23), and nausea (OR = 1.84, 95% CI: 0.72–4.72). The incidences of upper respiratory tract infection (OR = 0.79, 95% CI: 0.27–2.32), nasopharyngitis (OR = 0.40, 95% CI: 0.15–1.07), and urinary tract infection (OR = 1.82, 95% CI: 0.59–5.60) were generally lower in the etrasimod groups and no treatment-related serious infections were reported.

**Conclusion:**

This study demonstrates that etrasimod is effective in treating moderately to severely active ulcerative colitis with a favorable benefit-risk profile at week 12. Etrasimod shows promise as a potential first-line oral therapy for individuals suffering from this disease. Additional RCTs with larger sample sizes and longer observation periods are needed to confirm the sustained efficacy of etrasimod beyond the initial phase.

## 1 Introduction

Ulcerative colitis (UC) is a chronic, progressive, immune-mediated disease characterized by diffuse mucosal inflammation with a relapsing-remitting course. Elevated levels of inflammatory T cells in the gastrointestinal tract are the main characteristic of ulcerative colitis. The exact pathogenesis of UC is complex and has not been completely understood (Kobayashi et al., 2020). Disruption of the intestinal barrier was believed to be the initial cause of UC, inducing an inflammatory cascade that eventually leads to the chronicity of the disease. Multiple factors like genetic background, environmental factors, and mucosal immune dysregulation may contribute to the cause of UC pathogenesis. It is estimated that millions of people across all ages were affected by this disease, and an increasing trend in incidence and prevalence has been observed worldwide over these years (Kobayashi et al., 2020). The chronic relapsing nature of this disease imposes heavy burdens on the patients both physically and psychologically, severally reducing the patient’s quality of life or even leading to disability.

The current treatment for ulcerative colitis involves a combination of medications and non-medication therapeutics, depending on the severity of the disease, the extent of inflammation, and its evolution over time. Treatment initiation should not be postponed in moderate to severe cases to avoid delaying or disease progression. Conventional medical therapy typically includes aminosalicylates, corticosteroids, and immunomodulators, which aim to reduce inflammation and achieve symptom remission (Raine et al., 2022). Corticosteroids and biologics are preferred for acute treatment of UC while aminosalicylates were generally recommended as the first-line therapy for initial treatment and remission maintenance (Kobayashi et al., 2020; Siegel et al., 2020). Biologic agents (anti-integrin antibodies ustekinumab, TNF antagonist vedolizumab, etc.) and Janus kinase inhibitors (tofacitinib, etc.) are among the most effective therapeutics for UC (Feagan et al., 2013; Sandborn et al., 2017; Sands et al., 2019; Lasa et al., 2022). The development of novel therapeutic agents for UC in recent years has greatly expanded the therapeutic armamentarium for UC and provided patients with more treatment options than ever for improved treatment outcomes (Kobayashi et al., 2020; Hanzel et al., 2022; Abraham and Glassner, 2021). However, though these therapies provided effective and personalized treatment options for patients with UC, many patients still experience inadequate or unsustained response, disease relapse, inconvenience leading to poor adherence, and unacceptable adverse effects. These issues severely affect the patient’s quality of life or even lead to treatment discontinuation, indicating an unmet medical need for additional treatment options (Siegel et al., 2020; Sandborn et al., 2019; Bhattacharya and Osterman, 2020; Ma and Choi, 2022).

Moreover, with advances in medical treatment since the 2000s, the treatment goals of UC have shifted from resolving symptoms and achieving short-term improvement of patient’s quality of life to achieving endoscopic mucosal healing, aiming at reducing future relapse of disease and the need for treatment escalation, hospitalization, and surgery. This presents a great challenge for new drug research for UC, as well as biomarker identification that can precisely reflect the pathophysiology of the disease state and that can predict the efficacy and safety of a particular drug before administration.

S1PR are five G-protein-coupled receptor subtypes (S1PR1–5) that are highly expressed in T cells and are mainly involved in the modulation of the immune response, making it an ideal therapeutic target for treating immune-related diseases (Cyster and Schwab, 2012; Kihara et al., 2014; Lee et al., 2024). S1PR modulators sequester lymphocytes in the lymph nodes, inhibiting the migration of T cells from the lymph nodes to areas of inflammation. This helps to reduce the chronic inflammation process and has shown encouraging results in the treatment of Crohn’s disease (CD) and UC (Sukocheva et al., 2020; Bencardino et al., 2023). In clinical trials, etrasimod has been shown to significantly reduce symptoms and achieve endoscopic improvement in patients with moderate to severe UC who are intolerant or refractory to at least one conventional, biologic, or Janus kinase inhibitor therapy with an acceptable safety profile. These positive results highlight the potential of etrasimod as a valuable therapeutic option for patients with relapsing UC and led to its approval by the U.S. Food and Drug Administration (FDA) in October 2023 for adults with moderately to severely active UC.

Following ozanimod, etrasimod is the second selective S1PR modulator approved by the FDA for the treatment of UC. However, etrasimod is superior to ozanimod in its pharmacokinetic and drug metabolism properties. First, etrasimod does not require a slow titration over 7 days to reach the recommended maintenance dose, eliminating the potential delay in symptom relief for UC patients (Bencardino et al., 2023). Second, etrasimod directly targets S1PR to achieve clinical effects, while ozanimod relies on biotransformation steps to form several active metabolites, leading to a longer onset of action (Surapaneni et al., 2021). The elimination half-lives of ozanimod and its long-acting active metabolites were about 20–22 h and 10 days, respectively (Tran et al., 2020). As for etrasimod, the elimination half-life was about 29.7–36.4 h, and no major pharmacologically active metabolites were discovered (Lee et al., 2024). As a result, the median time to lymphocyte recovery for etrasimod and ozanimod after drug cessation was about 7 days and 1–2 months, respectively (Lee et al., 2024). The rapid onset and offset of action of etrasimod grants it a slight advantage over ozanimod in treatment selections and is the potential best-in-class S1PR modulator.

According to the literature, there have been published review articles evaluating S1PR modulators for the treatment of UC, and Suilik et al. performed a meta-analysis evaluating all doses of S1PR modulators ozanimod and etrasimod for both induction and maintenance therapy of UC (Suilik et al., 2023; Solitano et al., 2023). However, there is a lack of data analysis focusing solely on etrasimod at the recommended dose of 2 mg during the induction phase. In this article, we performed a meta-analysis based on available randomized controlled trials (RCTs) to systematically evaluate the efficacy and safety of etrasimod (2 mg) in the treatment of UC during the induction phase to provide a reference for its clinical application.

## 2 Materials and methods

### 2.1 Study search and selection

We have registered our protocol on The International Prospective Register of Systematic Reviews (PROSPERO) website (registration number: CRD42024574720). We performed a systematic search in the electronic databases including PubMed, Embase, the Cochrane Library, Clinical Trials, and the International Clinical Trials Registry Platform by using ‘Etrasimod’ or ‘Velsipity’ or ‘APD334’ as search terms for eligible studies from inception up to January 17, 2024. The search was limited to only studies published in English. The research was limited to RCTs comparing the efficacy of etrasimod with placebo or active comparators for moderate to severe UC. Thus, the inclusion criteria for this study encompassed patients with a diagnosis of moderate to severe UC; phase II or III RCTs intervention with etrasimod; intervention with the recommended dose of etrasimod (2 mg); comparison with another treatment drug or placebo; and reporting of efficacy and safety outcomes, including clinical remission, clinical response, achieved endoscopic improvement, histologic remission, and adverse effects (AEs). The *in vitro* study, pharmacokinetic/pharmacodynamic study, those without a comparator group, and non-RCTs were excluded from the analysis. Included RCTs were identified through a systematic literature review following the guidelines and recommendations of The Cochrane Collaboration. Two researchers screened and reviewed each study independently, and the inclusion of a study was decided by consensus between the two investigators. Any disagreement that happened in the process was resolved by consulting a third researcher. The reference lists in the included studies were also checked for potentially eligible studies. If an included study contained more than one dose of etrasimod, only data from patients taking the recommended dose of 2 mg were included for analysis. All the data were extracted from the included studies, including the authorship, year of publication, study design, study duration, study site, study population, interventions and comparators, clinical outcomes, and risk of AEs.

### 2.2 Outcome measurement

The study’s primary endpoint was the proportion of patients who achieved clinical remission at week 12. Key secondary endpoints included the proportion of patients with clinical response, endoscopic improvement, and histologic remission. The incidence of AEs, serious AEs (SAEs), and AE-related treatment discontinuation were statistically analyzed to evaluate the overall safety profile of etrasimod.

### 2.3 Data analysis

The Cochrane risk of bias tool was used to evaluate the quality of the included studies and their potential risk of bias. Two researchers independently reviewed all included studies and graded them as ‘low risk’, ‘high risk’, or ‘unclear risk’ based on the assessment items in the tool. Statistical analyses were conducted using Review Manager version 5.3. Pooled odds ratios (ORs) with a 95% credibility interval (CI) were used to compare the efficacy and safety of etrasimod with placebo or comparators. Study heterogeneity was assessed using the Chi-squared-based Cochran’s Q statistic and I^2^. Significant heterogeneity was considered when *P* < 0.10 or I^2^ > 50%. The fixed-effect model was employed for homogeneous data, while the random-effect model was utilized when heterogeneity was found to be substantial.

## 3 Results

### 3.1 Search and study characteristics

**FIGURE 1:**
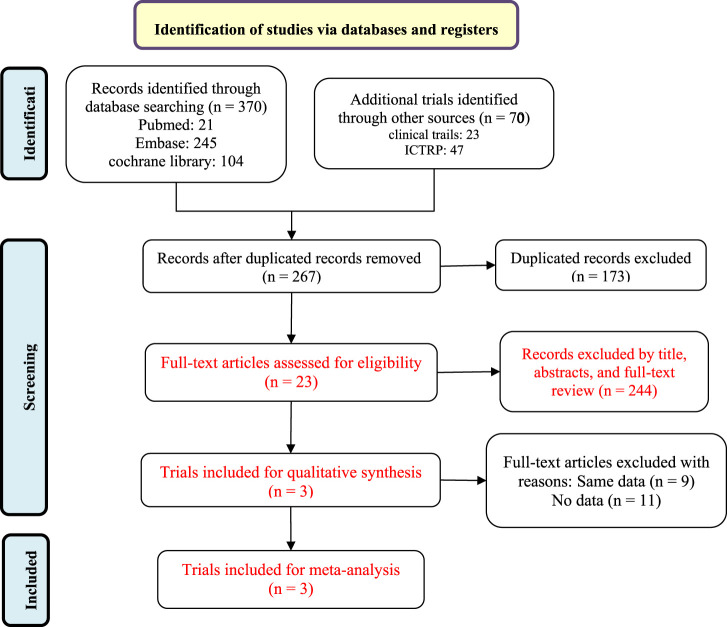
Flowchart of the study selection process.

A flow diagram of the study selection is presented in Figure 1. The initial search generated 440 articles, which, after removing 173 duplicates, left 267 articles for initial screening. Another 244 articles were excluded by title, abstracts, and full-text review, leaving 23 for eligibility assessment for qualitative synthesis. Three randomized controlled trials (RCTs) involving 943 patients met the inclusion criteria and were ultimately included in the systematic review and meta-analysis (Sandborn et al., 2020; Sandborn et al., 2023). All three studies were placebo-controlled, conducted between 2015 and 2021 in multiple countries. All trials employed a placebo-controlled, double-blind design. Sandborn’s study protocol (NCT02447302) consists of multiple dosing regimens and the group of patients taking the dose of 1 mg was excluded from the analysis (Sandborn et al., 2020). Excluding the group of patients taking etrasimod 1 mg in the NCT02447302 trial, a total of 891 participants were finally analyzed, with 581 receiving etrasimod (2 mg) and 310 receiving placebo. 507 (57%) patients were male and all were diagnosed with moderate to severe active UC. Details of included RCTs and characteristics of the included patients are presented in Table 1.

**TABLE 1:** Details of included RCTs and characteristics of the included patients.

Study	Intervention	Therapy duration	Study population	Study design	Male (%)	Age (years), Mean ± SD
NCT02447302	Etrasimod 1 mg (N = 52)	12-weeks	Adult outpatients with moderately to severely active ulcerative colitis	Phase 2 trial, proof-of-concept, double-blind, parallel-group study	30 (57.7)	43.2 ± 12.22
	Etrasimod 2 mg (N = 54)				27 (54.0)	40.4 ± 12.39
	Placebo (N = 50)				32 (59.3)	44.8 ± 14.85
NCT03996369	Etrasimod 2 mg (N = 238)	12-weeks	Adult outpatients with moderately to severely active ulcerative colitis	Randomised, multicentre, double-blind, placebo-controlled, phase 3 trials	135 (57%)	40.3 ± 13.5
	Placebo (N = 116)				73 (63%)	40.4 ± 13.3
NCT03945188	Etrasimod 2 mg (N = 289)	52-weeks	Adult outpatients with moderately to severely active ulcerative colitis	Randomised, multicentre, double-blind, placebo-controlled, phase 3 trials	152 (53%)	41.2 ± 14.0
	Placebo (N = 144)				88 (61%)	38.9 ± 14.0

**FIGURE 2:**
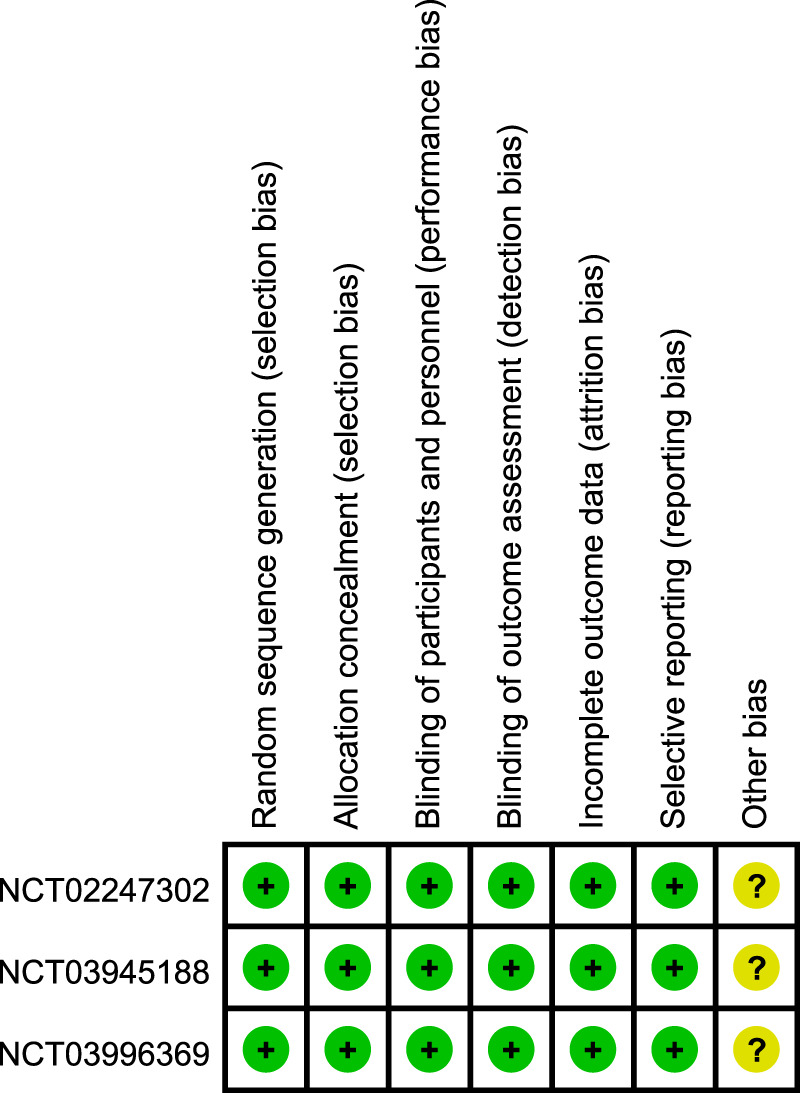
Risk of bias summary.

**FIGURE 3:**
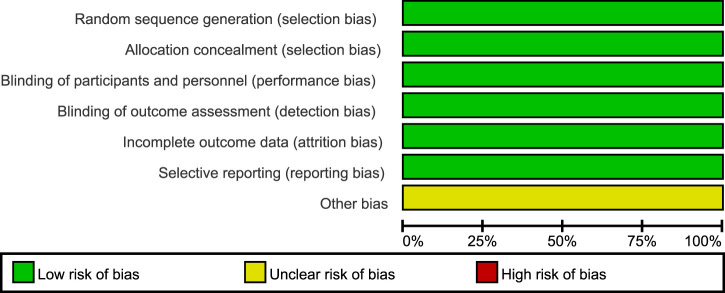
Risk of bias graph.

According to the Cochrane Collaboration tool for assessing the risk of bias, all three included trials were classified as having a low risk of bias, and eligible for meta-analysis (Higgins et al., 2019). Details of bias assessment are shown in Figures 2, 3.

### 3.2 The efficacy and safety of etrasimod for moderately to severely active ulcerative colitis

#### 3.2.1 Efficacy

**FIGURE 4:**
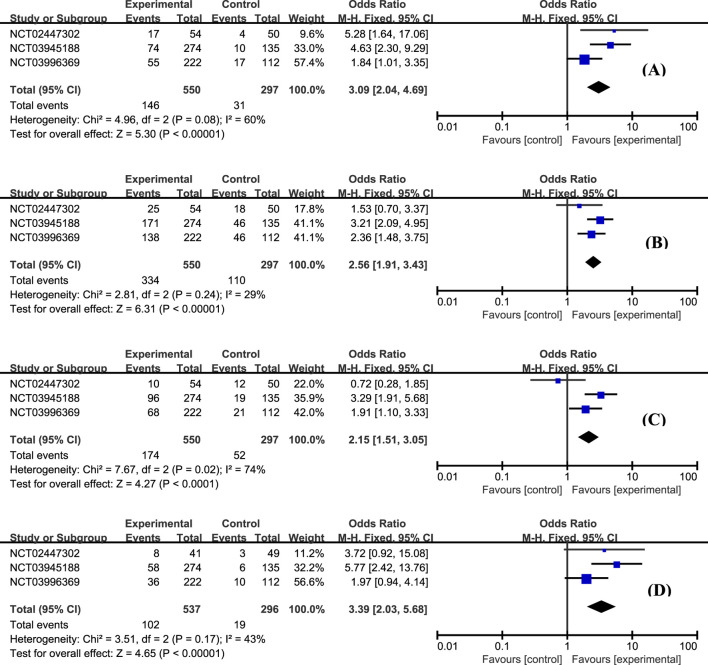
The proportion of patients achieving clinical remission **(A)**, clinical response **(B)**, endoscopic improvement **(C)**, and histologic remission **(D)** in the etrasimod and control group.

The efficacy data extracted from the included studies are presented in Figure 4. All three studies reported the outcome of clinical remission at week 12 (Sandborn et al., 2020; Sandborn et al., 2023). This meta-analysis revealed that the proportion of patients achieving clinical remission in the etrasimod group was significantly higher than placebo (Figure 4A, OR = 3.09, 95% CI: 2.04–4.69). A similar superiority was also observed for the secondary endpoints clinical response (Figure 4B, OR = 2.56, 95% CI: 1.91–3.43), endoscopic improvement (Figure 4C, OR = 2.15, 95% CI: 1.51–3.05), and histologic remission (Figure 4D, OR = 3.39, 95% CI: 2.03–5.68).

#### 3.2.2 Safety

**FIGURE 5:**
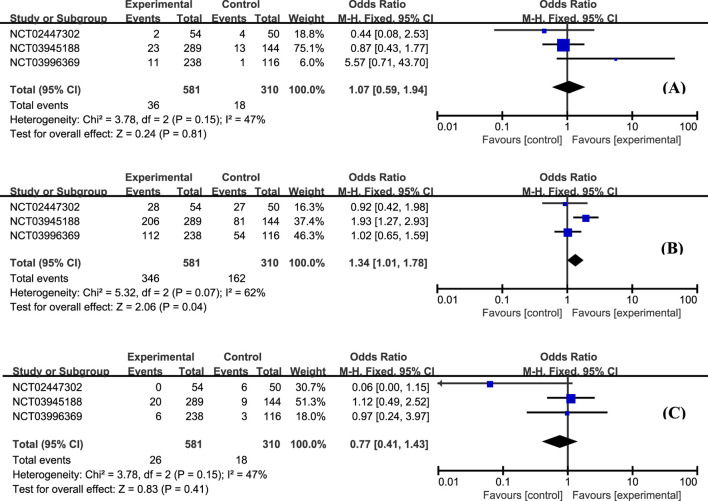
The proportion of patients with worsening UC **(A)**, occurrence of TEAE **(B)**, and serious TEAE **(C)** in the etrasimod and control group.

According to this analysis, the most common AE was worsening of UC, and no statistically significant difference was observed between etrasimod and control groups (Figure 5A, OR = 1.07, 95% CI: 0.59–1.94). Moreover, the overall proportion of patients with treatment-emergent adverse effects (TEAE) was similar between groups, and most of the TEAEs were mild to moderate severity (Figure 5B, OR = 1.34, 95% CI: 1.01–1.78). The occurrence of serious adverse effects was low and balanced between etrasimod and placebo groups (Figure 5C, OR = 0.77, 95% CI: 0.41–1.43).

**FIGURE 6:**
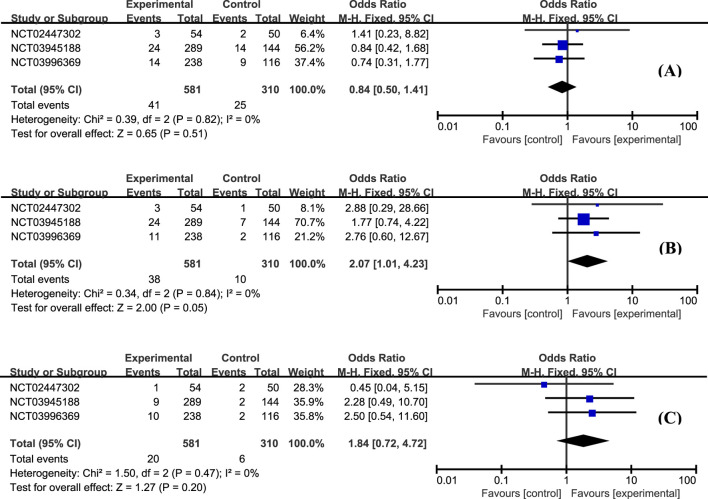
The proportion of patients with anemia **(A)**, headache **(B)**, and nausea **(C)** in the etrasimod and control group.

**FIGURE 7:**
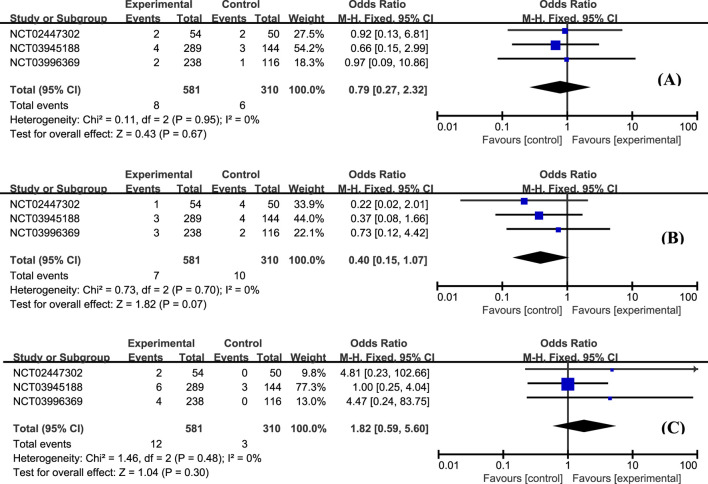
The proportion of patients with upper respiratory tract infection **(A)**, nasopharyngitis **(B)**, and urinary tract infection **(C)** in the etrasimod and control group.

The most frequently reported adverse effects in all studies included anemia, headache, and nausea. The proportion of patients with anemia was similar between groups (Figure 6A, OR = 0.84, 95% CI: 0.50–1.41). Patients with etrasimod had slightly higher odds of experiencing headache (Figure 6B, OR = 2.07, 95% CI: 1.01–4.23), and nausea (Figure 6C, OR = 1.84, 95% CI: 0.72–4.72). Considering the action mechanism of etrasimod, infection is a potential AE requiring particular concern. Upper respiratory tract infection (Figure 7A, OR = 0.79, 95% CI: 0.27–2.32), nasopharyngitis (Figure 7B, OR = 0.40, 95% CI: 0.15–1.07), and urinary tract infection (Figure 7C, OR = 1.82, 95% CI: 0.59–5.60) were the most reported infection cases, of which the incidences were generally low in all groups.

## 4 Conclusion

This study demonstrates that etrasimod is effective in treating moderately to severely active ulcerative colitis with a favorable benefit-risk profile at week 12. Etrasimod shows promise as a potential first-line oral therapy for individuals suffering from this disease. Additional RCTs with larger sample sizes and longer observation periods are needed to confirm the sustained efficacy of etrasimod beyond the initial phase.

## 5 Discussion

Our study revealed that etrasimod 2 mg significantly improved the proportion of patients achieving clinical remission, clinical response, endoscopic improvement, and histologic remission over placebo during the induction phases. Moreover, based on the positive results of Sandborn’s study, Vermeire et al. performed an open-label extension study evaluating the long-term safety and efficacy of the etrasimod 2 mg dose for up to 52 weeks (Vermeire et al., 2021). Consistent with the analysis results presented above, 64% of the 112 patients taking etrasimod had a clinical response, 33% were in clinical remission, and 43% had endoscopic improvement. More importantly, the proportion of patients who achieved clinical remission, clinical response, and endoscopic improvement at week 12 and maintained their respective response at the end of treatment was 60%, 85%, and 69% respectively. 54% of patients who initially showed a clinical response at week 12 achieved clinical remission at the end of treatment, illustrating a trend of continuous efficacy improvement. This implies that etrasimod may serve as an effective therapy not only during the induction phases but also during the maintenance phases, aligning with previous similar findings (Suilik et al., 2023).

The safety of etrasimod is a significant concern for supervisors, physicians, and patients due to the crucial roles S1PR plays in regulating multiple physiological activities in the body (Peyrin-Biroulet et al., 2017; Lasa et al., 2021). While early S1PR modulators have raised concerns about cardiovascular risks, the findings in this study indicate a relatively favorable safety profile of etrasimod. No serious cardiovascular events have been reported in this study, possibly due to the selective targeting of etrasimod toward S1PR subtypes 1, 4, and 5. This suggests that etrasimod may be suitable for patients with pre-existing cardiovascular comorbidities, which presents a considerable advantage in the selection of UC treatments. Moreover, data showed that the proportion of patients with TEAE, headache, nausea, and urinary tract infection was generally higher in the etrasimod group. But it should be noted that these events were either mild or moderate, localized, and did not lead to treatment discontinuation. No treatment-related serious infections were observed. More importantly, a pooled data analysis of clinical trials revealed that the safety profile of etrasimod did not change with the longer-term treatment of up to 2.5 years, demonstrating its superior safety over conventional therapies (Vermeire et al., 2023).

Furthermore, the convenient oral administration route of etrasimod may greatly improve the patient’s drug compliance, which is essential as UC treatments require long-term medication. Also, S1PR modulators are not immunogenic, which reduces the risk of immune response-related adverse effects commonly seen with biologics.

In general, the approval of etrasimod provided physicians with alternative induction therapy for UC with acceptable efficacy and safety profile. Etrasimod has the potential to be used as the first-line oral therapy for moderate-to-severe active UC treatments. However, it should be noted that the true clinical significance of drugs can be determined only by head-to-head comparisons and larger trials, and safety is also a significant concern for comparison. There is little comparative data between etrasimod, ozanimod, and other available drugs to date. More clinical data is needed to provide further insights about the positioning of etrasimod in the treatment algorithm of UC (Atreya and Neurath, 2023; Matsuoka and Hibi, 2023; Wils and Peyrin-Biroulet, 2023). With the rapidly emerging clinical data and therapeutic options approved for the treatment of UC, frequently updated systemic reviews and direct or indirect comparisons are necessary and may provide valuable guidelines in clinical settings (Lasa et al., 2022; Singh et al., 2020).

Though etrasimod showed superior efficacy in this study, it should be noted that there are still more than half of patients failed to achieve clinical remission or endoscopic improvements, and this was consistent with previous conventional therapies (Van Assche et al., 2016; Alsoud et al., 2021). An unmet medical need for more efficacious therapies for UC still exists. Additionally, the efficacy and safety data of etrasimod in real clinical settings are still missing, for example, there is still no data on their efficacy in patients with extraintestinal manifestations. Moreover, how and when to consider reducing or stopping treatment to minimize the risks, costs, and burdens to patients of prolonged drug therapy is also an important consideration and remains to be elucidated.

In addition, it is notable that bradycardia or sinus bradycardia was reported in patients receiving etrasimod treatment in this study, though most of the incidences were asymptomatic and resolved without pharmacological interventions (Wils and Peyrin-Biroulet, 2023). Considering the action mechanism of etrasimod, cardiovascular safety is an issue requiring particular concern. UC is a chronic condition that requires ongoing treatment and management. Long-term studies and real-world data are still needed to determine the sustainability of its benefits and the true risk profile of etrasimod in patients with UC.

In conclusion, while etrasimod has shown promise in improving outcomes for patients with ulcerative colitis, there are still gaps in our understanding of its real-world effectiveness and optimal treatment strategies. Addressing these knowledge gaps is essential for advancing the care and management of patients with this chronic condition.

## Data Availability

The original contributions presented in the study are included in the article/supplementary material, further inquiries can be directed to the corresponding authors.
